# Recent Advances in Therapeutic Approaches for Adult T-cell Leukemia/Lymphoma

**DOI:** 10.3390/v7122960

**Published:** 2015-12-14

**Authors:** Koji Kato, Koichi Akashi

**Affiliations:** Department of Medicine and Biosystemic Science, Graduate School of Medical Science, Kyushu University, Fukuoka 812-8582, Japan; akashi@med.kyushu-u.ac.jp

**Keywords:** adult T-cell leukemia/lymphoma, allogeneic hematopoietic stem cell transplantation, graft-versus-host disease, mogamulizumab, chemotherapy, antiviral therapy, J0101

## Abstract

Adult T-cell leukemia/lymphoma (ATLL) is a peripheral T-cell lymphoma caused by human T-cell leukemia/lymphoma virus type 1 (HTLV-1). ATLL occurs in approximately 3%–5% of HTLV-1 carriers during their lifetime and follows a heterogeneous clinical course. The Shimoyama classification has been frequently used for treatment decisions in ATLL patients, and antiviral therapy has been reportedly promising, particularly in patients with indolent type ATLL; however, the prognosis continues to be dismal for patients with aggressive-type ATLL. Recent efforts to improve treatment outcomes have been focused on the development of prognostic stratification and improved dosage, timing, and combination of therapeutic modalities, such as antiviral therapy, chemotherapy, allogeneic hematopoietic stem cell transplantation, and molecular targeted therapy.

## 1. Introduction

Adult T-cell leukemia/lymphoma (ATLL) was first described in 1977 by Uchiyama *et al.* [1], as a distinct clinical entity frequently observed in southwestern Japan. The causative agent of ATLL is the retrovirus human T-cell leukemia virus type I (HTLV-1) [2], which also causes several immune-associated diseases, including HTLV-1-associated myelopathy/tropical spastic paraparesis (HAM/TSP) [3]. ATLL develops in approximately 3%–5% of HTLV-1 carriers and has a dismal prognosis. However, the clinical manifestations and the course of disease in ATLL patients vary to a great extent. Therefore, recent efforts to improve treatment outcomes in ATLL patients have been focused on the development of prognostic stratification and therapeutic modalities. In this review, recent advances in ATLL treatment including antiviral therapy, chemotherapy, allogeneic hematopoietic stem cell transplantation (allo-HSCT), and molecular targeted therapy are discussed.

## 2. Diagnosis and Prognostic Factors for ATLL

ATLL diagnosis is based on clinical features, serum anti-HTLV-1 antibody, and ATLL cell morphology. The clonality of ATLL as a mature T-cell malignancy is confirmed by identification of the monoclonal integration of HTLV-1 proviral DNA in malignant cells by Southern blot analysis. The quantification of HTLV-1 integration site clonality has been recently developed through deep sequence analysis [4]. A high proviral load in HTLV-1 carriers is suggested to be associated with the development of ATLL, although HTLV-1 proviral load is not used as a diagnostic criterion of ATLL. In 1991, the Japan Clinical Oncology Group (JCOG) proposed the Shimoyama classification that defines four clinical subtypes: acute, lymphoma, chronic, and smoldering (Table 1) [5]. The classification is based on the presence of organ involvement, leukemic manifestation, high lactate dehydrogenase (LDH) and hypercalcemia that altogether reflect the prognosis and natural history of the disease. Chronic-type ATLL can be further divided into favorable and unfavorable types based on LDH, blood urea nitrogen, and albumin concentration. Further, acute, lymphoma, and unfavorable chronic types are defined as aggressive-type ATLL, while favorable chronic and smoldering types are defined as indolent-type ATLL [6]. For the last two decades, this clinical classification has been widely used as a guide in ATLL treatment.

**Table 1:** Diagnostic criteria and classification (the Shimoyama classification).

	Smoldering	Chronic	Lymphoma	Acute
Anti-HTLV-I antibody	+	+	+	+
Lymphocyte (×10^9^/L)	<4	≥4 ^†^	<4	*
Abnormal T lymphocytes	≥5%	+^‡^	≤1%	+^‡^
Flower cells with T-cell marker	Occasionally	Occasionally	No	+
LDH	≤1.5 N	≤2 N	*	*
Corrected Ca2^+^ (mEq/L)	<5.5	<5.5	*	*
Histology-proven lymphadenopathy	No	*	+	*
Tumor lesion				
Skin	§	*	*	*
Lung	§	*	*	*
Lymph node	No	*	Yes	*
Liver	No	*	*	*
Spleen	No	*	*	*
Central nervous system	No	No	*	*
Bone	No	No	*	*
Ascites	No	No	*	*
Pleural effusion	No	No	*	*
Gastrointestinal tract	No	No	*	*

HTLV-I, human T-lymphotropic virus type-I; LDH, lactate dehydrogenase; N, normal upper limit. * No essential qualification except terms required for other subtype(s); ^†^ Accompanied by T-lymphocytosis (3.5 × 10^9^/L or more); ^‡^ In case abnormal T-lymphocytes are less than 5% in peripheral blood, histology-proven tumor lesion is required; § No essential qualification if other terms are fulfilled, but histology-proven malignant lesion(s) is required in case abnormal T-lymphocytes are less than 5% in peripheral blood.

Although the prognosis of aggressive ATLL is dismal, there is marked diversity among patients. A prognostic index for acute- and lymphoma-type ATLL (ATL-PI) has been proposed based on a retrospective analysis of 807 newly diagnosed patients between January 2000 and May 2009 in Japan [7]. Ann Arbor stage (I–II *vs.* III–IV), Eastern Cooperative Oncology Group performance status (ECOG PS; 0–1 *vs.* 2–4), age, serum albumin, and soluble interleukin-2 receptor (sIL-2R) were statistically significant prognostic factors. A simplified ATL-PI was as follows: prognostic score; +2 (Ann Arbor stage = III or IV); +1 (ECOG PS > 1); +1 (age > 70); +1 (albumin < 35 g/L); and +1 (sIL2R > 20,000 U/mL). Scores from 0 to 2 were categorized as low risk, 3 to 4 as intermediate risk, and 5 to 6 as high risk. The median overall survival times (MST) were 16.2 months in low-risk patients, 7.0 months in intermediate-risk patients, and 4.6 months in high-risk patients. However, the Shimoyama classification and ATL-PI were established based on retrospectively collected data; thus, the patient characteristics, such as the type of treatment and prognostic factors, were not comparable between groups. The JCOG prognostic index (JCOG-PI) has recently been established based on data from 276 patients with aggressive ATLL in three prospective JCOG trials, which identified poor PS and hypercalcemia as significant prognostic factors [8]. In patients with corrected calcium of <2.75 mmol/L and a PS of 0 or 1 (moderate risk), the MST and five-year overall survival (OS) were 14 months and 18%, respectively; in patients with corrected calcium of ≥2.75 mmol/L and/or a PS of 2–4 (high-risk), the MST and five-year OS were eight months and 4%, respectively. The JCOG-PI may be useful in identifying aggressive ATLL patients with dismal prognosis. Assessment by both ATL-PI and JCOG-PI will certainly be useful in identifying patients with extremely poor prognosis among aggressive ATLL cases. In addition, several biomarkers, such as CC chemokine receptor 4 (CCR4), lung resistance-related protein, and p53 mutations, have been reported [9,10]; however, so far, prognostic models and biomarkers that are able to identify patients who may not need allogeneic hematopoietic stem cell transplantation (allo-HSCT) do not exist. Thus, further investigation is needed to establish robust prognostic models.

## 3. Treatment for ATLL

The treatment strategy for ATLL patients is based on the clinical subtype according to the Shimoyama classification [5,9,10,11]. The watchful waiting strategy or interferon-α (IFN-α)/zidovudine (AZT) are usually reserved for patients with indolent-type ATLL, whereas chemotherapy, allo-HSCT, and newer therapeutic agents are preferred for patients with aggressive-type ATLL. In Europe and the USA, antiviral therapy using IFN-α/AZT is the standard treatment for leukemic-type ATL. Importantly, a subset of patients with indolent type ATLL experience skin lesions that can be treated with either skin-directed therapy, such as topical steroids, ultraviolet light, and radiation, or systemic therapy, such as steroids, oral retinoids, or single agent chemotherapy. The current treatment strategies are summarized in Table 2.

**Table 2:** Treatment strategy for adult T-cell leukemia/lymphoma (ATLL).

**1. Indolent-type ATLL: Smoldering- or favorable chronic-type**
(1) Watchful waiting for asymptomatic patients
(2) Interferon-α (IFN-α)/zidovudine (AZT) or watchful waiting for symptomatic patients
(3) Skin lesion:
Local therapy; Topical steroids, Ultraviolet light, Radiation
Systemic therapy; Steroids, Oral retinoids, Single agent chemotherapy
**2. Aggressive-type ATLL: Unfavorable chronic-, lymphoma- or acute-type**
(1) Chemotherapy:
VCAP-AMP-VECP
CHOP or less-toxic regimen for elderly patients
(2) VCAP-AMP-VECP + mogamulizumab
(3) Allogeneic hematopoetic stem cell transplantation (allo-HSCT)
(4) IFN-α/AZT (except for lymphoma-type)
**3. Relapse or refractory ATLL**
(1) Mogamulizumab
(2) Allo-HSCT
(3) New agents under clinical trial:
Brentuximab vedotin, Bortezomib, Lenalidomide, Panobinostat, Forodesine
Pralatrexate, Denileukin diftitox
(4) Vaccine (autologous dendritic cells with tax-peptide)

VCAP-AMP-VECP: vincristine, cyclophosphamide, doxorubicin, and prednisolone (VCAP); doxorubicin, ranimustine, and prednisolone (AMP); and vindesine, etoposide, carboplatin, and prednisolone (VECP). CHOP: doxorubicin, cyclophosphamide, vincristine and prednisone.

### 3.1. Interferon-α and Zidovudine

Combined IFN-α/AZT has been reported effective as an ATLL treatment [12]. An international consensus meeting recommended the IFN-α/AZT combination or watchful waiting in patients with indolent ATLL [13]. A meta-analysis of 254 ATLL patients, including 116 patients with acute type, 100 with lymphoma type, 18 with chronic type, and 11 with smoldering type, reported that patients with acute-, chronic-, and smoldering leukemic-type ATLL had better outcomes with first-line antiviral therapy alone, whereas chemotherapy was more effective in patients with lymphoma-type ATLL [14]. Specifically, the five-year OS of patients with chronic and smoldering indolent-type ATLL was 100% with antiviral therapy. Overall, the meta-analysis concluded that first-line antiviral therapy improved the survival of ATLL patients. JCOG has started a phase III study comparing IFN-α/AZT with watchful waiting to determine any potential benefits from early intervention in patients with indolent-type ATLL.

### 3.2. Chemotherapy

To date, several chemotherapy regimens such as CHOP (cyclophosphamide, doxorubicin, vincristine, and prednisolone) and EPOCH (VP-16, prednisolone, vincristine, cyclophosphamide, doxorubicin) have been assessed in patients with aggressive ATLL [15]. Particularly, since 1978, the JCOG-Lymphoma Study Group (JCOG-LSG) has played a central role in the development of chemotherapy regimens for ATLL patients [16]. Most recently, JCOG-LSG conducted a randomized clinical trial (JCOG9801) to compare the modified LSG15 (mLSG15) regimen with a biweekly CHOP regimen (CHOP14) in untreated patients with aggressive-type ATLL [17]. The original LSG15, *i.e.*, VCAP-AMP-VECP, regimen sequentially consisted of vincristine, cyclophosphamide, doxorubicin, and prednisolone (VCAP); doxorubicin, ranimustine, and prednisolone (AMP); and vindesine, etoposide, carboplatin, and prednisolone (VECP). The mLSG15 regimen replaced one course of VCAP-AMP-VECP from the original LSG15 regimen with intrathecal administration of methotrexate and prednisone as prophylaxis against central nervous system relapse. The CHOP14 regimen consisted of doxorubicin, cyclophosphamide, vincristine and prednisone. The complete remission (CR) rate of 40% with mLSG15 was significantly better than the CR rate of 25% observed with CHOP14. The three-year OS rates were 24% and 13% for mLSG15 and CHOP14 regimens, respectively; however, the difference between the two groups was not statistically significant. In Japan, mLSG15, which has been recommended as the first-line treatment for aggressive-type ATLL at an international consensus meeting [13], is feasible as a standard regimen in patients with aggressive ATLL who are less than 56 years old. However, the mLSG15 regimen is not routinely available throughout the world due to restricted use of the medications such as ranimustine and vindesine. In addition, the 13-month MST with mLSG15 is unsatisfactory compared with other hematological malignancies. Currently, there are no salvage chemotherapy options established for patients with relapsed or refractory ATLL, and further investigation is needed.

### 3.3. Allogeneic Hematopoietic Stem Cell Transplantation

Allo-HSCT has become an important curative treatment modality in patients with aggressive-type ATLL during the last decade; however, intensified chemotherapy and autologous HSCT has not been successful [18]. Since Utsunomiya *et al.* [19] reported successful outcomes in 10 ATLL patients receiving allo-HSCT in 2001, the number of ATLL patients receiving allo-HSCT has been increasing. In earlier cases, patients received allo-HSCT almost always from human leukocyte antigen (HLA)-matched related donors (MRD) with full-intensity conditioning (FIC) [19,20,21]. Along with the well-developed Japanese marrow donor program/cord blood bank and improvements in supportive care, unrelated bone marrow (UBM) and umbilical cord blood (UCB) have been increasingly used as alternative donor sources [22,23,24,25]. In addition, with the introduction of reduced-intensity conditioning (RIC), the number of allo-HSCT has been steadily increasing [26]; so far, *i.e.*, by 2015, more than 1500 ATLL patients have received allo-HSCT in Japan. According to donor sources, the three-year OS rate was 41% in patients with MRD and 39% with UBM in a nationwide survey [27]. In contrast, the outcomes of allo-HSCT from UCB were unsatisfactory with the three-year OS rate of 17%, partially due to the overlap of the study period that was around 2005, with the developmental phase of allo-HSCT from UCB in adult patients. The recently updated data on ATLL patients showed that the three-year OS of allo-HSCT from UCB remained at 20.6% compared with the three-year OS rates of 34.4% and 37.1% with allo-HSCT from MRD and UBM, respectively [28]. It is certainly challenging to directly compare outcomes from different donor sources because the graft source selection is strongly influenced by donor availability. Nevertheless, the outcomes of CBT in ATLL patients continue to be unsatisfactory due to the high transplant-related mortality (TRM) of 46.1%. Novel interventions will be required, particularly during the early phase, to reduce TRM and control for graft-*versus*-host disease (GVHD) in patients receiving allo-HSCT from UCB [29,30]. Although most allo-HSCT outcomes have been reported in Japanese ATLL patients, the European Group for Blood and Marrow Transplantation’s Lymphoma Working Party has recently shown similar results with allo-HSCT in ATLL patients in western countries [31]. However, most of these findings were based on retrospective analysis and patients with heterogeneous backgrounds, including chemosensitive and refractory diseases at transplantation. An ongoing prospective study is assessing the safety and efficacy of RIC followed by allo-HSCT in ATLL patients who achieved remission at transplantation and are stratified according to donor source. Okamura *et al.* [32,33] first reported that the five-year OS of allo-HSCT from MRD was 34% in this prospective study. Other prospective trials assessing RIC followed by allo-HSCT from UBM and UCB are also ongoing. In particular, the three-year OS of allo-HSCT from both MRD and UBM was approximately 30%, indicating that allo-HSCT is a curative treatment. However, survival rates of allo-HSCT have not dramatically improved during the last decade. The major risk factor affecting the survival of ATLL patients receiving allo-HSCT is disease status at transplantation. Based on the incidence rate of ATLL, about 80%–90% of ATLL patients are not able to receive allo-HSCT mostly due to disease resistance to chemotherapy. Therefore, further efforts are needed to increase the response rate prior to allo-HSCT.

### 3.4. Novel Agents

One recent promising therapeutic progress in ATLL is the introduction of mogamulizumab for the treatment of patients with relapsed or refractory ATLL. Mogamulizumab is an anti-CCR4 monoclonal antibody that markedly enhances antibody-dependent cellular cytotoxicity through high-affinity binding to effector cells. CCR4 is selectively expressed on regulatory T-cells and T-helper type 2 (Th2) cells and is expressed on the surface of most ATLL cells. In addition, CCR4 expression is highly associated with poorer prognosis. Based on the data of tolerability in a phase I study, [34] a phase II study for relapsed ATLL was subsequently conducted wherein 1.0 mg/kg of mogamulizumab as a single agent was intravenously administered once a week for eight weeks [35]. The overall response rate (ORR) was reported to be 50%. The median progression-free survival (PFS) was 5.2 months, and the OS was 13.7 months. Based on these results, mogamulizumab use was approved in Japan on March 2012, although mogamulizumab use is not available outside of Japan except in clinical trials. A second, randomized phase II clinical trial in newly diagnosed patients with aggressive ATLL has recently demonstrated that the CR rate was higher at 52% with mLSG15 in combination with mogamulizumab compared to 33% with mLSG15 alone, whereas there was no statistical difference in survival [36]. Since these responses were reported not to be long-lasting, allo-HSCT is still needed for a cure even with the introduction of mogamulizumab. Concurrently, mogamulizumab has been expected to serve as a bridge to transplantation to achieve better disease control during allo-HSCT and to improve survival. However, care should be taken with the use of mogamulizumab in patients with allo-HSCT as CCR4 is expressed not only on tumor cells but also in normal regulatory T-cells and Th2 cells. In a non-transplantation setting, severe skin reactions, such as Steven–Johnson syndrome have been reported [37]. Moreover, rare side effects after the administration of mogamulizumab, such as diffuse panbronchiolitis [38] and colitis [39], have recently been reported. In patients with allo-HSCT, the mogamulizumab treatment may accelerate GVHD by eradicating T-cells. Therefore, the safety and benefits of mogamulizumab both before and after allo-HSCT should be evaluated, and further clinical experience and accumulation of data is necessary [40,41]. To that end, a multicenter prospective observational study is now underway to evaluate the safety and efficacy of mogamulizumab use in ATLL patients with relapsed or refractory disease even after allo-HSCT. In addition to mogamulizumab, clinical trials are underway to determine the efficacy of other novel agents, including brentuximab vedotin, bortezomib, lenalidomide, panobinostat, forodesine, pralatrexate, and denileukin diftitox [9,42]. So far, it seems to be difficult to improve the dismal prognosis of ATLL through these novel agent monotherapies. However, in future study, it is important to determine how new agents should be combined with conventional chemotherapy and allo-HSCT.

### 3.5. Immunotherapy

Allo-HSCT is a curative treatment approach in ATLL patients, partly through its graft-*versus*-ATLL (GvATLL) effect as described before. Grade I/II (mild to moderate) acute GVHD has been shown to be associated with improved survival rates [43,44]. The discontinuation of immunosuppressive agents or donor lymphocyte infusion was also effective in ATLL patients who relapsed even after allo-HSCT [45,46]. In addition, Tax- or HBZ-specific T-cells have been shown to play an important role in inducing a potent GvATLL effect [47,48]. The vaccine, which was developed by pulsing Tax peptide into autologous dendritic cells, was effective in ATLL patients [49]; a clinical trial is ongoing for the evaluation of this vaccine with mogamulizumab in Japan.

## 4. Conclusions

The prognosis of ATLL patients, despite an increase in the variety and potency of therapeutic options, has undeniably remained poor, and many obstacles still exist [50]. In future efforts, through studies that uncover the molecular mechanisms underlying ATLL, new treatment protocols integrating antiviral therapy, chemotherapy, allo-HSCT, and molecular targeted agents with optimized dosing and timing need to be developed.
